# Aggregation Properties of Albumin in Interacting with Magnetic Fluids

**DOI:** 10.3390/ijms221910734

**Published:** 2021-10-03

**Authors:** Elena N. Velichko, Elina K. Nepomnyashchaya, Maksim A. Baranov, Alexey N. Skvortsov, Ivan V. Pleshakov, Ge Dong

**Affiliations:** 1 Institute of Electronics and Telecommunications, Peter the Great St. Petersburg Polytechnic University, 195251 Saint Petersburg, Russia; 2 Institute of Biomedical Systems and Biotechnology, Peter the Great St. Petersburg Polytechnic University, 195251 Saint Petersburg, Russia; askvortsov@spbstu.ru; 3 Ioffe Institute, 194021 Saint Petersburg, Russia; ivanple@yandex.ru; 4 School of Aerospace Engineering, Tsinghua University, Beijing 100084, China

**Keywords:** protein–nanoparticle interaction, molecular electronics, magnetic fluid, self-organization, nanoparticle sizing, dynamic light scattering

## Abstract

In this study, interactions of Fe_3_O_4_ magnetic nanoparticles with serum albumin biomolecules in aqueous solutions were considered. The studies were conducted with the laser correlation spectroscopy and optical analysis of dehydrated films. It was shown that the addition of magnetite to an albumin solution at low concentrations of up to 10^−6^ g/L led to the formation of aggregates with sizes of up to 300 nm in the liquid phase and an increase in the number of spiral structures in the dehydrated films, which indicated an increase in their stability. With a further increase in the magnetite concentration in the solution (from 10^−4^ g/L), the magnetic particles stuck together and to albumin, thus forming aggregates with sizes larger than 1000 nm. At the same time, the formation of morphological structures in molecular films was disturbed, and a characteristic decrease in their stability occurred. Most stable films were formed at low concentrations of magnetic nanoparticles (less than 10^−4^ g/L) when small albumin–magnetic nanoparticle aggregates were formed. These results are important for characterizing the interaction processes of biomolecules with magnetic nanoparticles and can be useful for predicting the stability of biomolecular films with the inclusion of magnetite particles.

## 1. Introduction

The study of the interaction of biomolecules with nanoparticles is of vital importance for medicine, biophysics, and biochemistry. Recently, the interactions of biomolecules with metal and metal oxide nanoparticles have attracted attention in the development of a new field of biomolecular electronics. Modern semiconductor electronics have nearly reached the limits of their miniaturization capabilities. The geometrical dimensions of transistors are already less than several nanometers, and their further reduction is hindered by fundamental quantum-mechanical limitations. In this regard, the development of molecular electronics seems to be an urgent task. To solve it, the unique properties of organic molecules and their hybrids with solid-state electronics can be used [1]. Such systems are of a miniature size, and the surface density of their device elements can be increased to 10^13^–10^14^ units per cm^2^ for a nanometer layer [2,3]. To create organic–inorganic hybrid materials, complete information about their properties, which are essential for the formation of substrates and active layers, is required [4].

Among these properties is the ability of biomolecules to self-assemble. Due to cooperative intra- and intermolecular physicochemical interactions, biomolecules ideally reproduce a spatial structure that is unique for each type of biomolecule. Such properties are widely used in different medical applications [5], such as drug delivery system design [6]. Moreover, complexes and self-organized films formed from biomolecules on a substrate can be used to store, transform, and transmit energy and information with an extremely high density in time and space [7]. Such films are used for most prototypes of biomolecular nanodevices [8,9,10,11] in electronics, optics, and biotechnology [12,13]. Electronic elements, such as field-effect transistors [14,15,16], solar batteries [17], sensors [18], and biocompatible electrodes, have already been developed and are now successfully functioning [19,20,21,22,23,24,25].

Immobilized biomolecules with the addition of nanoparticles are often used to increase the speed, sensitivity, and selectivity of biomolecular devices, as well as to protect them against degradation [26]. Thus, the interaction processes of nanoparticles with biomolecules [27], as well as their effects on the activity preservation of the biological molecules immobilized on the electrode surfaces, require comprehensive study [28,29]. In our work, special attention was given to the study of hybrid materials based on biomolecules in combination with magnetic nanoparticles. Such materials are of interest for both fundamental science and applications in devices [30,31] due to their nonlinear optical properties, which can be adjusted by controlling the magnetic field [32,33]. Magnetic nanoparticles are already widely used in medicine [34,35,36,37], but their influence on biomolecule self-organization processes is still under discussion [38,39,40,41].

In order to use magnetic fluids in biomolecular electronics, it is important to understand the properties of magnetization, aggregation and sedimentation stability, and biomolecule interactions, including the latter’s influence on self-organization in thin macromolecular films [42]. In our work, we studied the clusterizaton processes of magnetic nanoparticles with biomolecules in a liquid solution and their influence on the self-organization of biomolecules during isothermal dehydration, leading to the formation of thin biomolecular films on substrates.

To detect clustering at the nanometer scale, we used laser correlation spectroscopy (LCS), which can be used to assess the parameters of molecular clusters, such as size distribution and concentration. Among existing experimental methods for nanoparticle control, the LCS technique was chosen as a very convenient and powerful tool for nanoparticle–biomolecular binding studies (see Table 1).

The influence of clustering on microscale self-organization in thin biomolecular films was investigated via the computer analysis of film images. Microscale self-organization, as a direct method of assessing the binding of biomolecules with nanoparticles during condensation, was used to obtained information on the magnetic sensitivity of proteins.

This study is of great theoretical and practical importance for the development of hybrid nanoelectronic devices of the next generations.

## 2. Materials and Methods

### 2.1. Laser Correlation Spectroscopy

The clustering dynamics in liquids play important roles in the formation of aggregates with predictable physical properties, which is important for the development of biomolecular electronics. Dynamic light scattering is among the most effective methods for analyzing clustering in liquids. It uses the parameters of scattered field fluctuations, which makes it possible to overcome classical diffraction limits and extend the range of investigated sizes down to 1 nm [54,55].

**Figure 1:**
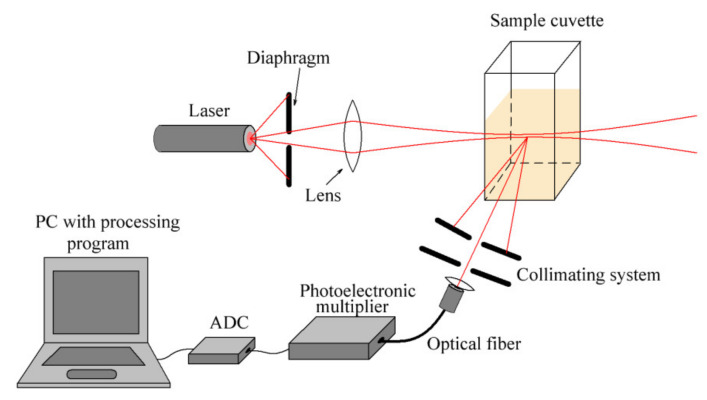
Scheme of the laser correlation spectrometer.

We used an original modification of the classical approach of dynamic light scattering measurements by laser correlation spectroscopy, an approbation and detailed description of which were presented in [56,57,58]. Our main modification consists of an original scheme shown in Figure 1 and the data processing algorithm adopted for the investigation of polydisperse solutions. This method is especially effective for studies of aggregation properties in nanosystems, including organic–inorganic hybrid molecular systems characterized by multicomponent compositions.

The experiment can be briefly described as follows. A sample (3 mL volume) was placed in rectangular sectioned glass cell and irradiated with coherent laser radiation (633 nm wavelength; 10 mW), and the power of the light field scattered by the sample was recorded at an angle of 90 degrees using photoelectronic multiplier, ADC, and computer. The length of scattering volume in the solution was selected by the collimating system and was equal to 6.5 mm. The signals represented the dependence of the recorded light power on time. The analysis of these signals was carried out on a computer, which calculated and analyzed the autocorrelation function. More information about processing procedure can be found in [58].

**Figure 2:**
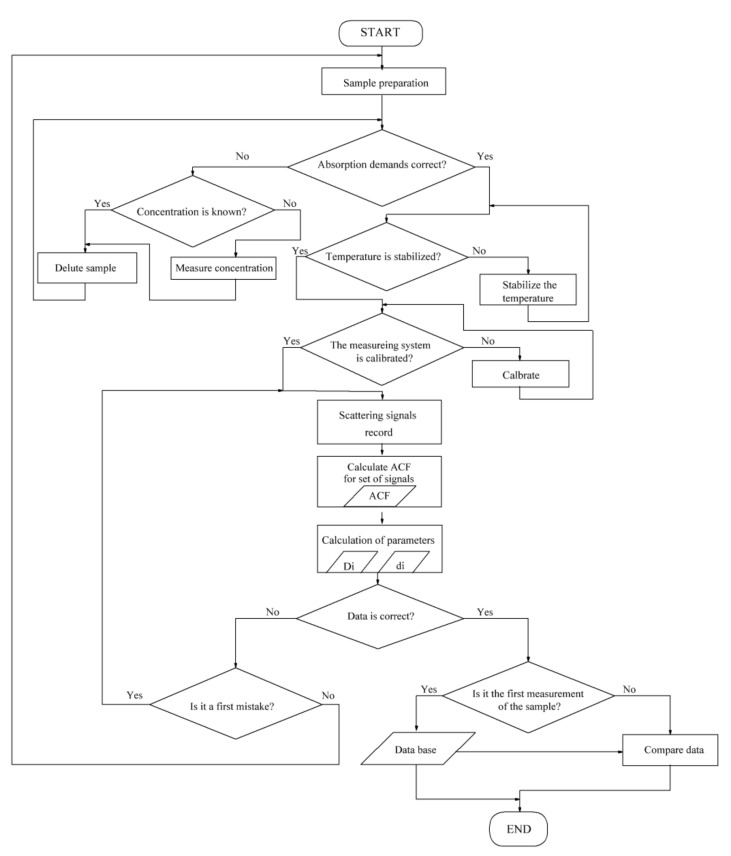
Algorithm for conducting the LCS experiment.

The general algorithm of the laser correlation spectroscopic experiment is shown in Figure 2. Within the framework of the experiment, a liquid sample was prepared. In this case, the sample was a solution of biological molecules with the addition of magnetic nanoparticles. Descriptions of solutions and preparation techniques are given in Section 2.3. To estimate the clustering parameters by laser correlation spectroscopy, it was important to use nonabsorbing samples in the spectral wavelength range used in the experiment. This requirement was checked before the measurements. Thermal stabilization was provided during all measurements. The device was calibrated with reference samples.

According to the theory of dynamic scattering, the experimental correlation function $g\left(\tau \right)$ can be approximated as:

(1) $$g\left(\tau \right)=\sum_{i}F\left(q,\;D_{i}\right)e^{-q^{2}D_{i}\tau },$$

where *F*(*q*, *D_i_*) is the distribution of scattering power as a function of the diffusion coefficients and the angle of detection (θ), q is the wave vector given by the expression $q=\frac{4\pi n_{0}}{\lambda_{0}}\sin\left(\frac{\theta }{2}\right)$, $\lambda_{0}$ is the light wavelength,$\;n_{0}$ is the refractive coefficient, and *Di* is the translational diffusion coefficient [59], which can be calculated as follows:

(2) $$D_{i}=k_{b}T/3\pi \eta d_{i},$$

where *k_b_* is the Boltzmann constant, *T* is the temperature, *¦Ç* is the viscosity of the liquid, and *d_i_* is the particle diameter. Thus, the LCS data processing ultimately yields an *F*(*q*, *D_i_*) distribution.

### 2.2. Isothermal Dehydration

To prepare samples of thin biomolecular films, the method of isothermal dehydration was chosen. The technology of this method is based on the law of the evaporation of liquid molecules, which is generally determined by the Clausius–Clapeyron equation:

(3) $$n_{a}=bT^{1/2}\exp\left(-l_{a}/k_{b}T\right),$$

where $n_{a}$ is the number of particles evaporating from a unit area per unit time, $l_{a}$ (*J*) is the particle evaporation work, *T* (*K*) is the temperature, *k_b_* (*J/K*) is the Boltzmann constant, and *b* $(K^{-1/2}$) is the thermal evaporation constant.

In general, the mass transport of water from liquid surfaces is affected by the temperature, composition of the gas phase, activity of surface substances, bulk rheological properties and kinetics of vapor transport, and heat flux through the surface area, as well as the geometry of experimental conditions [60,61,62]. The preparation of thin biomolecular films, which are characterized by a small thickness compared to their area, can also be described by the theory of the evaporation process.

As a result of the isothermal dehydration of biomolecular solutions, periodic patterns with a wide range of shapes are typically formed in the film. The structure shape is influenced by various parameters of the initial solutions of biological molecules, as well as the parameters of the dehydration experiment [63,64]. Due to the complex composition of biological fluids and a variety of physical, chemical, mechanical, and other processes, the mechanisms of the formation of specific structures during evaporation still lack a quantitative theory [65]. The formation of logarithmic spiral cracks is associated with the directional propagation of the mechanical pressure front [66]. The heterogeneity of surface coatings is caused by interfacial dynamic instabilities due to surface tension gradients, which are called Marangoni instabilities [67,68].

The formation processes of structures in films can be classified as examples of self-organization. Since the type of structures formed in thin films is determined by the properties of the biomolecular system, it seems possible to evaluate the properties of a biomolecular film by studying the structures formed in the film.

In our paper, we focused on revealing properties such as the stability of the biomolecular film or, in other words, its ability to maintain integrity under low mechanical stress. An important factor that influences stability is the adhesion of biomolecular films to their substrate. Stability also depends on many other factors such as the substrate material, residual humidity, concentration of peptides or other molecules in the film, the plasticizer type, and the presence of salt ions. The control of such a number of experimental conditions is a complicated task. Therefore, it was important to check the stability of each produced film. As shown by preliminary experimental studies, the stability of biomolecular films produced by dehydration from water solutions correlates with the formation of self-organized spiral structures. The structures can differ in shape, number of turns, outer cracks, and inner core diameters.

In our work, an experimental study of the formation of self-organized patterns in thin biomolecular films was carried out. Liquid solutions of biomolecules were placed in 20 mm Petri dishes and dried for 48 h at a temperature of 308 K, normal atmospheric pressure, and a humidity of 20 ± 1%. To dry the films, a TS-1/80-SPU thermostat with forced air circulation was used.

The images of the obtained films were recorded by using an Olympus CX 43 optical microscope with PlanC N 40× objective, aperture 0.10 (Olympus Corp., Tokyo, Japan) and an Altami UHCCD05000KPA USB camera with SONY ICX282AQ sensor (Altami LLC, St. Petersburg, Russia) with a resolution of 1280 × 980 and a depth of 24 bits. The spectral range of detection was 380–650 nm. Altami Studio software was used to measure the sizes of aggregates of magnetic nanoparticles in the film. We also calculated various geometric parameters of structures (including the number of spirals) by using the software developed by the authors [69].

### 2.3. Test Samples

Human serum albumin protein (HSA, Biotest Pharma GmHb, Dreieich, Germany) with an initial concentration of 200 mg/mL was selected as the main molecule of interest.

To prepare the experimental samples, an aqueous solution of albumin in distilled water with a concentration of 50 mg/mL was used, because this is the minimum protein concentration required to achieve adhesion to an inorganic wettable substrate, which is significant for the creation of three-layer sandwich systems of the substrate–film–metal type for modeling and developing functional devices of biomolecular electronics [29,70].

The magnetic fluid (MF) used in our study was an ion-stabilized aqueous colloid of magnetite (Fe_3_O_4_) provided by the Department of Colloidal Chemistry, St. Petersburg State Technological Institute. The diameter of solid phase nanoparticles was about 10 nm, which is typical of magnetic fluids prepared by the methods described in [71]. Their spontaneous clustering (still present in insignificant quantities in the systems similar to that considered in [72]) was prevented by the formation of an electric double layer by decreasing pH and inducing a positive charge at the oxide surface. The required parameters of the aqueous medium were achieved by adding hydrochloric acid until reaching a concentration of H^+^ ions approximately equal to 10^−5^ mol/L, which corresponded to pH ≈ 5.

The initial concentration of the solid phase was 0.8 mg/mL. For the study, the MF was diluted with distilled water until optical transparency was achieved. Solutions with concentrations of 1–10^−5^ mg/L were prepared. The samples were subjected to ultrasonic treatment for at least 10 min immediately before measurements or subsequent dilution. The ultrasound frequency was 42 kHz at a source power of 50 watts.

To analyze the effect of magnetic nanoparticles on the clustering and self-organization of the protein, as well as on the parameters of the biomolecular film formed from the solution, mixtures of MF with albumin solutions were prepared. As the MF volume was lower than 1.2% of the mixture volume, the concentration of biomolecules in all samples could be considered similar and equal to 50 mg/mL (~0.75 mmol/L). After adding the magnetic fluid to the biomolecule solution, it was carefully mixed by slowly flipping the cuvette with the prepared sample for several (5–10) min to achieve homogeneity.

All the samples were prepared at room temperature. Experimental studies were carried out immediately after the preparation of the samples.

## 3. Results

### 3.1. Assessment of Aggregate Sizes

**Figure 3:**
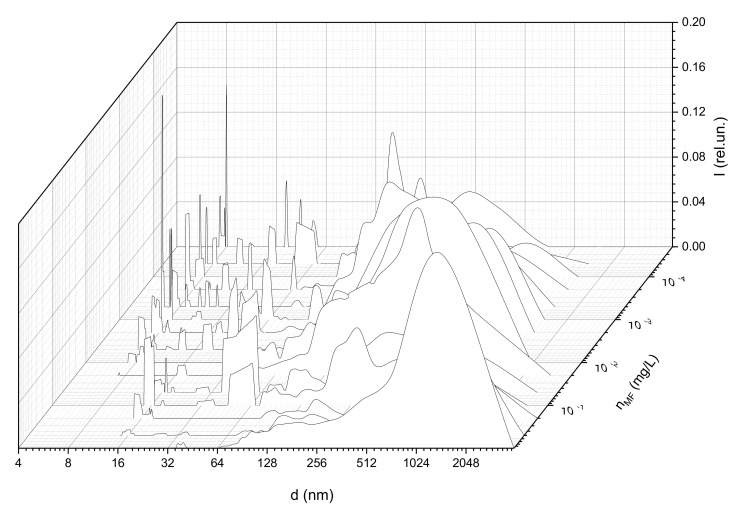
Size distribution of structures in albumin solutions with the addition of magnetic fluid at various concentrations.

Figure 3 shows the size distribution of structures in water solutions of albumin (50 mg/mL) with the addition of magnetic fluid at various concentrations (from 2·10^−5^ to 1 mg/L). In the graph, n_MF_ is the magnetic fluid concentration in the solution, d is the structure diameter, and I is the relative scattering intensity. The temperature of this and following LCS experiments was stabilized and equal to 300 °K. Measurements were organized under normal pressure in a dark room.

**Figure 4:**
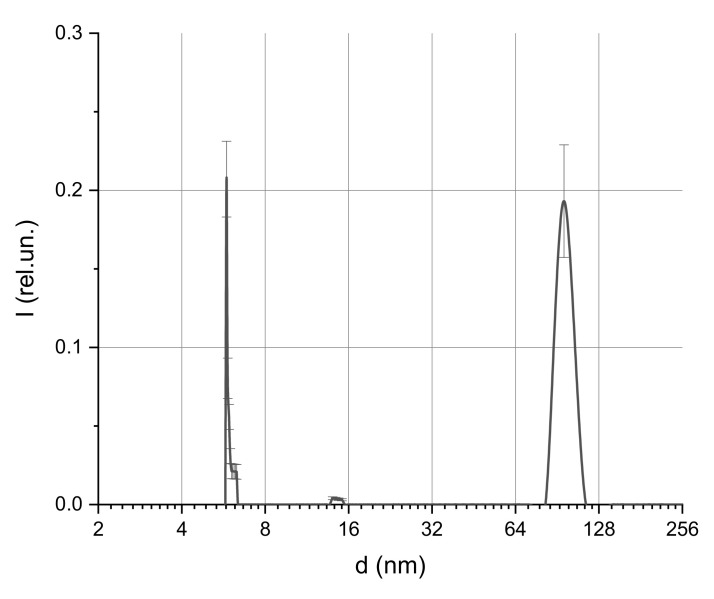
Size distribution of albumin protein in solution with a concentration of 50 mg/mL.

At low MF concentrations in the solution, individual particles with sizes of about 6 nm, which corresponded to single protein molecules, were detected. In addition, aggregates with sizes of 70–100 nm, presumably corresponding to albumin aggregates, were observed. This conclusion was based on preliminary experiments aimed to estimate the structure sizes in the pure albumin solution with a concentration of 50 mg/mL (Figure 4).

**Figure 5:**
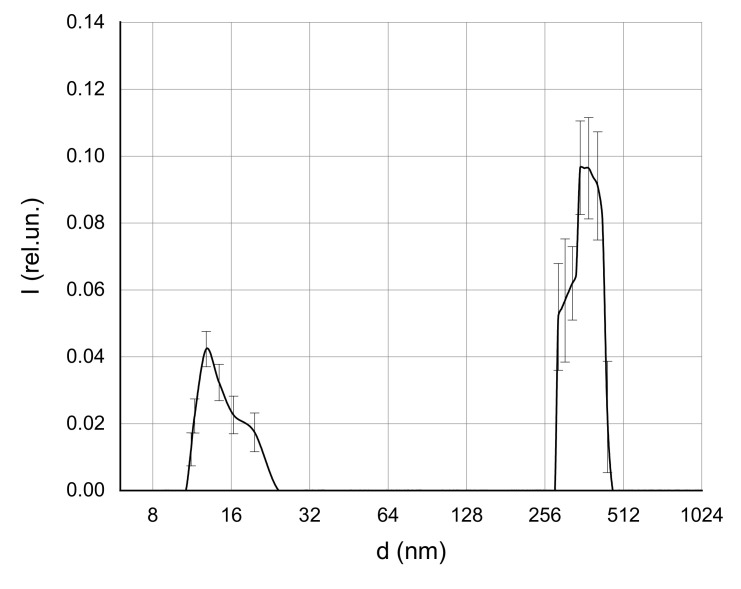
Size distribution of magnetic nanoparticles in MF solution.

The detected particles of 10–30 nm in size corresponded to single nanoparticles of a magnetic fluid. These particles were found in the study of pure MF solutions. Figure 5 presents the sizes of scatterers in MF with a concentration of 1 mg/L. The solution also contained larger aggregates with sizes of up to 500 nm, which corresponded to the aggregates of magnetic nanoparticles formed upon dilution. This effect was observed earlier and was described in [58].

**Figure 6:**
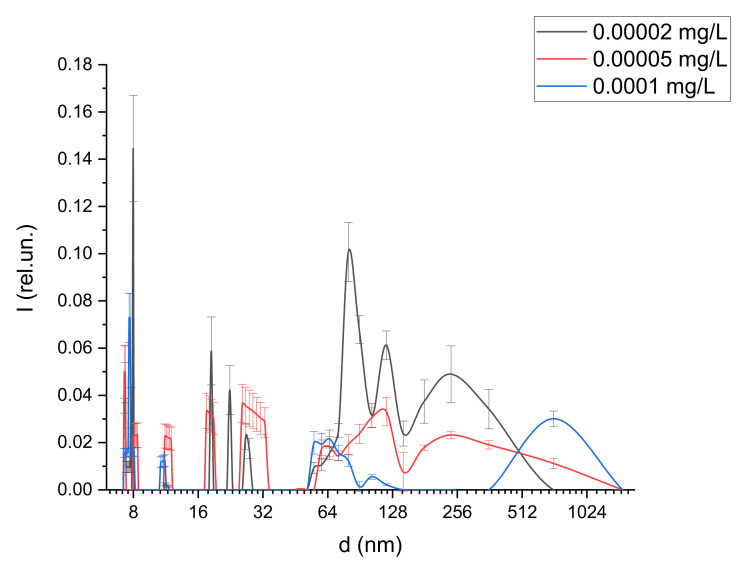
Size distribution of structures in albumin solutions with the addition of magnetic fluid at low concentrations.

Note that the addition of magnetite at a concentration of less than 0.1 µg/L had a weak effect on the sizes of protein aggregates. As the concentration increased from 0.1 µg/L, an additional peak with an average size of more than 500 nm appeared in the distribution (Figure 6); this indicates the aggregation process. The size of these aggregates indicated that they were composed of clusters containing bound albumin protein molecules and magnetic nanoparticles, since such aggregates were not found in the pure albumin or MF solutions.

**Figure 7:**
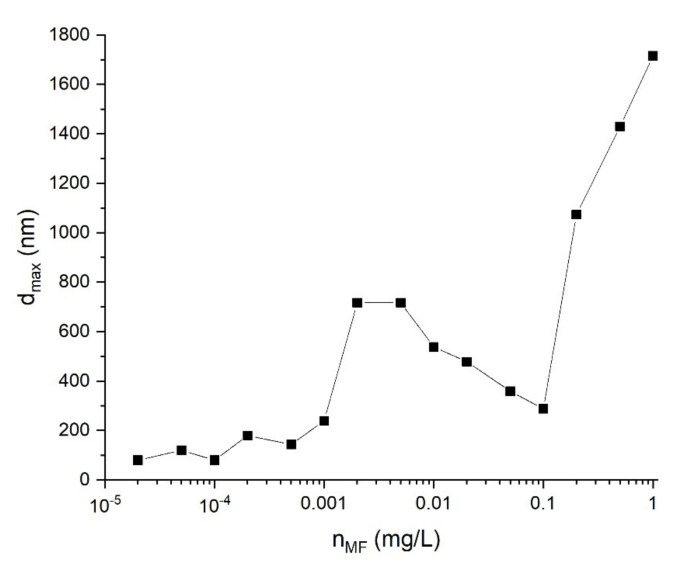
Dependence of the typical size of aggregates in albumin/magnetic fluid mixture on magnetic fluid concentration. d_max_ is the particle size corresponding to the largest mode of the intensity distribution.

The width of this peak and the position of its maximum value gradually increased (up to a typical size of 800 nm) as the concentrations of the magnetic fluid increased from 0.1 to 2 µg/L (see Figure 7, which presents sizes of aggregates with maximal relative concentration calculated from Figure 5). At MF concentrations of 2–100 µg/L, this trend was reversed—the average size of aggregates decreased and reached ~250 nm at an MF concentration of 100 µg/L. At the same time, aggregates with sizes of about 800 nm were still observed in the solution, but their relative concentration was lower than that of smaller aggregates. As the MF concentration increased to above 0.1 mg/L, the average size of the aggregates increased to 1000 nm and more. The growth in the size of aggregates in this case could be associated with the aggregation of magnetic fluid particles, both with albumin and among themselves.

The spatial structure of dehydrated protein films on a dielectric substrate, prepared by isothermal drying from an aqueous solution of albumin with various concentrations of magnetic nanoparticles, was experimentally studied.

**Figure 8:**
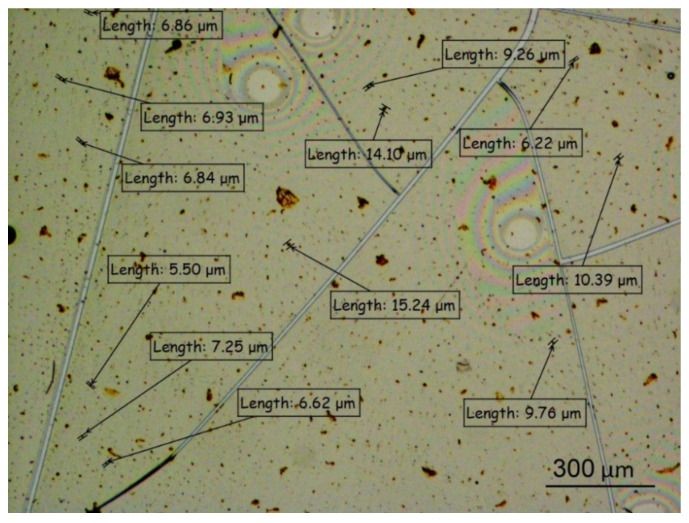
Photo of a dehydrated film obtained from an albumin solution (50 mg/mL) with the addition of MF at a concentration of 1 mg/L (center of the film).

The formation of aggregates of magnetic nanoparticle at high MF concentrations was confirmed by studies of dehydrated films. Figure 8 shows an image of the central part of albumin protein film (with initial concentration of 50 mg/mL in water) with the addition of magnetic fluid at a concentration of 1 mg/L. The obtained images showed the presence of magnetic nanoparticle aggregates of various sizes of 10 or more microns. Detected aggregates were studied by optical microscopy; their sizes are shown in Figure 8.

The size of aggregates dramatically increased (in comparison with Figure 7) in the process of dehydration due to the clusterization effects caused by changes of concentration during liquid phase vaporization. These effects usually occurred in the dried films of the nanoparticles and revealed the tendency of nanoparticles to form aggregates in solution.

Thus, it was established that aggregates consisting of biomolecules and metal oxide nanoparticles are formed in protein solutions containing magnetic fluids. The tendency of albumin to form aggregates with nanoparticles makes it possible to draw conclusions regarding the effects of nanoparticles on the properties of the protein itself, including its ability to self-organize. To test these assumptions, the following studies of albumin self-organization in the process of its dehydration and the formation of a molecular film were carried out.

### 3.2. Estimation of the Number of Structures in Dehydrated Films

As already noted, preliminary studies [73] showed that the size, number, and frequency of structures formed in molecular films during their dehydration correlate with film stability. Thus, the more stable the molecular film, the larger the number of spiral structures formed at its edges. Film stability is an necessary parameter for the formation of active biomolecular layers on a substrate.

**Figure 9:**
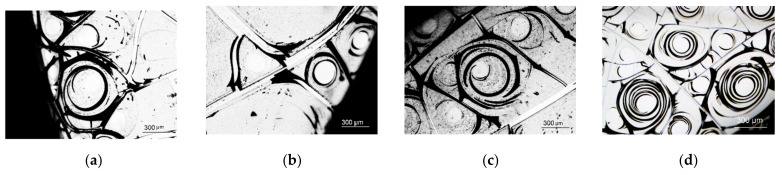
Examples of structures formed in albumin solutions (50 mg/mL) at different MF concentrations: (**a**) n_MF_ = 5 × 10^−5^ mg/L, (**b**) n_MF_ = 5 × 10^−2^ mg/L, (**c**) n_MF_ = 1 mg/L, and (**d**) pure albumin.

Here, we considered spiral structures, formed at the edges of a 20 mm Petri dish (a ring with about 3 mm of thickness). At the center areas of dishes, no self-organized structures were formed (see Figure 8). The density, size, and formation of the spiral structures varied depending on the magnetic fluid concentration. Figure 9 shows examples of the self-organized structures formed at the dish edges for different MF concentrations.

**Figure 10:**
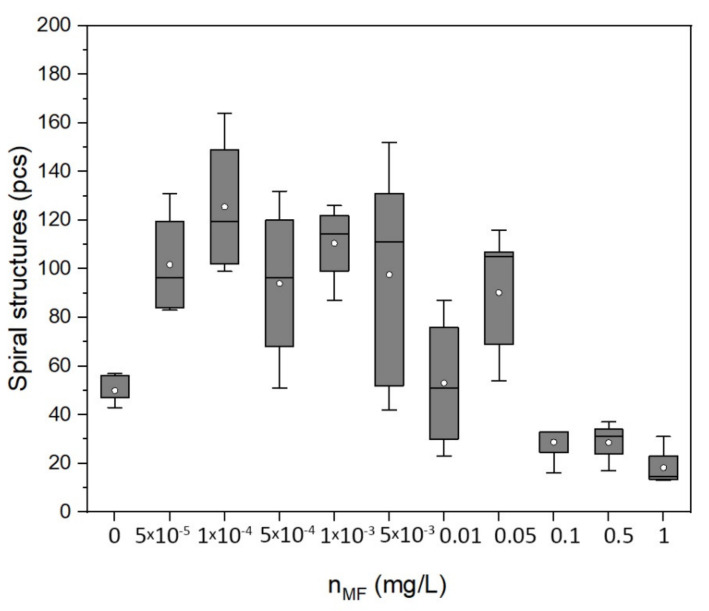
Dependence of the number of spiral structures on the concentration of MF in the albumin protein solution (quartile plots; point indicates mean value).

To characterize the process of protein self-organization and assess the effect of MF on the self-organization of albumin during its dehydration, the parameter of a total number of formed spiral structures in the film was chosen. Figure 10 shows the dependence of the number of formed spiral structures in the film (N) on the concentration of MF (n_MF_) calculated from pictures of dehydrated films.

It can be seen from the figure that the number of spiral structures increased to 150–160 as the concentration of magnetite in the protein solution increased from 0 to 10^−4^ mg/L. At concentrations above 0.05 mg/L, the number of spiral structures began to decrease with increasing magnetite concentration, reaching typical values of about 10.

## 4. Discussion

As already noted, the stability of molecular films has a critical effect on their applicability for the purposes of biomolecular electronics [29,70,74,75]. Since the current general trend towards the miniaturization of electronic components has led to the creation of thinner molecular films, the problem of their stability has become more urgent [76,77]. In previous work, the authors showed that as film thickness decreases, its stability is influenced by fluctuation forces [29,70,75]. The possibility of such an influence was predicted on the basis of previous studies [74]. The authors theoretically showed the effect of increasing the stability of peptide films with added nanoparticles—both single and deposited on a dielectric substrate.

Preliminary studies showed that in thin biomolecular films prepared by the method of isothermal dehydration, the effects of self-organization lead to the formation of a special film morphology containing periodic spiral structures [63]. In this case, the nature of the structures formed, namely their size, density, and number, correlated with the film stability [73,78]. In addition, preliminary data suggest that the character of the formed films is also influenced by the clustering parameters of molecules in the initial solutions [63].

In our study, the influence of the presence of magnetic nanoparticles on the morphology of periodic structures and the stability of protein films was investigated. The experimental studies revealed a strong influence of magnetite nanoparticles of various concentrations on the aggregation processes in protein solutions. The formation of protein–magnetite aggregates was observed. At the same time, the relative concentration of these aggregates turned out to be maximal at magnetite concentrations 1–100 µg/L. A further increase in the magnetite concentration led to the aggregation of magnetic nanoparticles not only with proteins but also with each other (Figure 7 and Figure 8).

When a small MF volume was mixed with an HSA solution, the MF was strongly diluted. As a result, its pH increased from 5 to values typical of HSA solutions (7.1–7.2 based on the results of direct pH measurements for individual samples). Under these conditions, HSA molecules (pI 4.7) are negatively charged, while Fe_3_O_4_ (pI 7.9 [79]) should theoretically have a weak positive charge [80]. Thus, both electrostatic interactions favoring the HSA binding to the surface of nanoparticles and an increase in the pH of the mixture decrease the aggregative stability of nanoparticles, which was observed here in the initial and final regions of the dependence of the size of HSA complexes with magnetic nanoparticles (Figure 7). In [80], the existence of a significant effect of pH on the adsorption of albumin protein on magnetic particles was confirmed; the maximum adsorption was detected around pH = 4.64. In [81], the influence of MF concentration on the amount of aggregated protein was reported; the general trend showed an increase of protein to Fe_3_O_4_ bounds with increasing amounts of nanoparticles in solution. Nevertheless, we detected a descending segment (Figure 7), which indicated a stabilizing effect of HSA molecules on the MF dispersion in a certain concentration range. One of the reasons for this may be the HSA binding to the magnetite surface due to non-Coulomb interactions. However, this fact requires further research. Similar results were presented in [82]. The albumin protein was used to stabilize magnetic nanoparticles from strong aggregation. The sizes of aggregates in the albumin–MF mixture were around 400 nm, while bare MF nanoparticles without stabilization formed aggregates with sizes of more than 600 nm. In [83], an increase in the size of albumin–magnetic nanoparticle aggregates compared to that of pure MF in the presence of high MF concentrations was shown.

The observed effect of magnetic nanoparticle aggregation with albumin protein on self-organization during the formation of a molecular film showed that the addition of low concentrations of magnetic fluid (5 × 10^−5^–5 × 10^−3^) mg/L to the protein solution in dehydrated films led to increases in the number of spiral structures compared to a reference samples with no added magnetite. This was a rather strong effect if we consider that at such concentrations of MF, there were 10^8^–10^10^ protein molecules of comparable size per magnetic nanoparticle (MF nanoparticle: ~10 nm; the albumin molecule was roughly elliptical: the smaller axis was 3.8 and the larger axis was 13–15 nm). On the contrary, an increase in the concentration of magnetite above 0.1 mg/L led to a decrease in the number of spiral structures in the film and an increase in the average size of aggregates.

Such a structure of protein films can be explained using the hypothesis of the formation of spiral structures partially described in [73]. Helical structures can be formed from gas bubbles dissolved in a protein solution. The release of air bubbles to the surface causes the stretching of the dried protein film and the formation of spiral cracks upon further drying. Magnetic nanoparticles immersed in a drying film also become points of inhomogeneity. A course initiation of spiral cracks is most likely to occur at the point of inhomogeneity, and the number of structures increases with increasing the MF concentration. However, the subsequent growth of spiral cracks requires a relatively uniform film. Therefore, it is possible that in the presence of a large number of large magnetic nanoparticle aggregates, the number of spirals formed decreases.

Summing up our experimental results on the interaction of proteins and magnetic particles, we can conclude that when low concentrations of magnetic fluid were added to protein solutions, biomolecule–magnetic oxide aggregates were formed. This was accompanied by the formation of a larger number of high-stability spiral structures in the dehydrated protein film. However, as the concentration of the magnetic fluid increased up to 1 mg/L, the HSA solution was unable to counteract the aggregation of magnetic particles, so aggregates that most likely contained both protein and magnetic particles were formed. This led to an increase in the solution viscosity and a decrease in its homogeneity while the number of spiral structures decreased.

The results of our optical microscopy suggested that the maximum stability of biomolecular films can also be observed at low concentrations of magnetic particles. At concentrations over 0.1 mg/L, the peeling of protein films from the substrate was observed.

Thus, correlations between the dynamics of the formation of protein films and the magnetic fluid concentration in the initial solution were revealed. The influence of magnetic nanoparticles on the stability of biomolecular films obtained by isothermal dehydration from albumin protein solutions was experimentally confirmed. We detected the effect of the increased stability of HSA–MF films at relatively low MF concentrations. Biomolecular films with an increased stability are in very high demand for potential use as functional elements of biomolecular electronics. At the same time, the obtainment of the film’s superparamagnetic properties [84,85,86,87] has opened up new fields of bioelectronic applications in spin electronics and magnetic resonance imaging where biofilms had yet to be used. Metal–protein bindings lead to the formation of a specifically oriented layer that can be considered to be a charge carrier for transistor elements. Peptide films with embedded metal oxide nanoparticles, especially those containing nanoparticles with magnetic properties, open up new interesting possibilities for applications in organic electronics and nanotechnology.

## 5. Conclusions

This paper presents a study of the aggregation of albumin molecules with magnetite nanoparticles in an aqueous solution. Characteristic features of aggregation with the formation of clusters with diameters of about 800 nm were revealed upon the addition of low concentrations (up to 0.1 mg/L) of an Fe_3_O_4_ solution. During the formation of dehydrated films from solutions, the addition of and a slight increase in the concentration of magnetic nanoparticles (up to 0.1 mg/L) led to an increase in the stability of the biomolecular film on the dielectric surface. With a further increase in the concentration of magnetic nanoparticles in the initial solution, the effects of the formation of clusters of magnetite particles were observed. These led to the formation of large structures in the solution and a gradual decrease in the stability of the formation of morphological structures in the dehydrated film obtained from such a solution.

The observed results showed that the stabilizing effect of albumin–magnetic nanoparticles mixture can occur in low MF concentrations from 10^−6^ to 10^−4^ g/L. Such concentrations allow for the formation of stable protein films with magnetic properties determined by magnetic fluids. Experimental results showing the aggregation of albumin protein with nanoparticles of ion-stabilized magnetic fluids at low concentrations are presented in our paper for the first time. Our unique results showed the violation of the linear character of the dependance of the BSA–MF aggregate size on MF concentration. Additionally, the stability of molecular films with embedded nanoparticles increased with increases in MF concentration in a certain range. A correlation was found between the stability of the film, the number of spiral structures formed in it, and the aggregate sizes. The obtained results enable the prediction of the stability of biomolecular films with the inclusion of magnetite particles.

The approaches presented in this paper have made it possible to study the effects of the interactions of biomolecules with nanoparticles, as well as the dynamics of their clustering. The described self-organized biomolecular layers of proteins can be considered a basis for the development of components of biomolecular electronics.

## Figures and Tables

**Table 1:** Methods of biomolecule–nanoparticle binding studies.

Method	Applications
Transmission electron microscopy	Measurements of the average diameter of nanoparticles; biomolecules with nanoparticle aggregates [43,44].
NMR spectroscopy	Site-specific measurements of protein–nanoparticle binding [45]; detection of the structures and interactions of biomolecules that are bound to material surfaces [46,47].
Isothermal titration calorimetry	Detection of kinetic and equilibrium binding affinities; study of association reactions [43].
Surface plasmon resonance	Characterization of protein−protein and nanoparticle−protein interactions; determination of absolute values of the affinity and kinetic constants [48].
UV–Vis absorption spectroscopy	Detection of conformational changes of biomolecules in interaction with nanoparticles [49]
Fluorescence analysis	Detection of conformational changes around Trp residues [49]; determination of specific binding sites [48].
Microscale self-organization	Visualization of the dynamics of protein condensation [50,51]; detection of binding [52].
Laser correlation spectroscopy	Control of binding of biomolecules and nanoparticles in liquids; detection of aggregation processes in time [43,53].

## Data Availability

Not applicable.
